# Effects of fluoride on *in vitro* hydroxyapatite demineralisation analysed by ^19^F MAS-NMR

**DOI:** 10.3389/fdmed.2023.1171827

**Published:** 2023-05-24

**Authors:** Bajram Ferizoli, Alexander J. Cresswell-Boyes, Paul Anderson, Richard J. M. Lynch, Robert G. Hill

**Affiliations:** Dental Physical Sciences Unit, Centre for Oral Bioengineering, Institute of Dentistry, Barts and the London School of Medicine and Dentistry, Queen Mary University of London, London, United Kingdom

**Keywords:** 19F MAS-NMR, fluorapatite, hydroxyapatite, fluoride, demineralisation, remineralisation

## Abstract

**Introduction:**

Fluoride plays a major role in inhibiting enamel dissolution and promoting fluorapatite formation. Porous hydroxyapatite (HAP) discs can be used as an enamel analogue in artificial demineralisation/remineralisation studies.

**Method:**

The aim of the study was to monitor the fluoride-mineral phases formed on HAP surfaces as a function of fluoride concentration ([F^−^]) under demineralising conditions, using ^19^F magic angle spinning nuclear magnetic resonance (MAS-NMR) spectroscopy, and compare the results with a previous study using an enamel substrate. Porous HAP blocks were immersed in demineralisation solutions (0.1 M acetic acid, pH 4.0) with increasing [F^−^] (0–1450 ppm).

**Results:**

At below 50 ppm [F^−^], ^19^F MAS-NMR showed fluoride-substituted apatite formation; above 50 ppm [F^−^], calcium fluoride (CaF_2_) was formed in increasing proportions. These results mirrored those of previous similar studies with an enamel substrate. Further increases in fluoride caused no further measurable reduction in demineralisation but increased the proportion of CaF_2_ formed. The total calcium concentration [Ca] and total phosphorus [P] concentrations in the solution were measured by inductively coupled plasma atomic emission spectroscopy. At high fluoride concentrations, the solution total [P] increased, and the molar Ca:P ratios decreased to values consistent with the formation of CaF_2_. However, Ca:P ratios found at low [F^−^] were higher than those in the previous enamel study and consistent with the formation of a partially fluoridated apatite.

**Conclusions:**

Under demineralising conditions, CaF_2_ formed on HAP at an [F^−^] of 50 ppm and above, whereas fluoridated apatite formed at an [F^−^] below 50 ppm. The results were consistent with those obtained when an enamel substrate was used.

## 1. Introduction

Dental caries remains a major problem in adults and is especially prominent in children (1–3). The most effective measures for preventing tooth decay involve the use of fluoride, either added to the water supply or incorporated into toothpaste, or the topical application of agents such as silver diamine fluoride (SDF) (4). Despite numerous studies in the literature, the exact aetiology of the action of fluoride on reducing demineralisation is not fully understood, although it is thought to involve the acid dissolution of hydroxyapatite (HAP) and the reprecipitation of a less soluble fluorapatite (FAP) or a highly fluoride-substituted HAP (5–8).

HAP is commonly used as an enamel analogue within *in vitro* studies for understanding caries or erosion (9–14). Mohammed et al. (15) investigated the demineralisation of enamel blocks immersed in caries-simulating 0.1 M acetic acid at pH 4.0 with different fluoride concentrations ([F^−^]). The formation of FAP was determined by ^19^F magic angle spinning nuclear magnetic resonance spectroscopy (MAS-NMR). In addition, the calcium and phosphorus concentrations in the demineralising solutions were measured using inductively coupled plasma optical emission spectroscopy (ICP-OES). The results showed that at low [F^−^] fluoridated apatite formed, while on the other hand, at 45 ppm [F^−^] and above, calcium fluoride (CaF_2_) formed. It is worth commenting that commercially available European toothpaste typically contains 1,450 ppm [F^−^], while prescription toothpaste can range as high as 23,000 ppm. Further, some in-office topical agents, such as SDF, can contain up to 51,013 ppm (16).

Solid-state ^19^F MAS-NMR is a powerful tool for investigating fluoride uptake and FAP formation. ^19^F has a natural abundance of virtually 100% and is a spin half nucleus, which ensures that measurements are relatively rapid to perform. ^19^F MAS-NMR measurements are easy to perform, provided care is exercised to eliminate ^19^F signals from the instrumentation, for example from the MAS-NMR probe itself. ^19^F spectra can be obtained for samples containing relatively little fluoride. In addition, ^19^F MAS-NMR is extremely sensitive and can be used to distinguish directly between calcium FAP and fluorite. ^19^F solid-state MAS-NMR detects all fluorine nuclei present, whether crystalline, amorphous, or adsorbed. All F nuclei in a sample can be detected (17). Recently, Gao et al. (18) showed the ^19^F signal in mixed fluorohydroxyapatites (FHAP) varied with the fluorine content, which potentially enables ^19^F MAS-NMR to determine the fluorine content from the observed ^19^F chemical shift.

Despite this, relatively few studies have employed ^19^F MAS-NMR in the oral healthcare and dental research literature. The emphasis has been on identifying the fluoridated apatite and CaF_2_ formed on or in tooth surfaces using indirect chemical techniques to measure acid- and alkali-desorbable fractions (19–23). Solid-state ^19^F MAS-NMR has been previously used to characterise fluoride–HAP interactions. Yesinowski and Mobley (24) demonstrated the ability of this technique to distinguish between FAP [Ca_10_(PO_4_)_6_F_2_,], FHAP [Ca_10_(PO_4_)_6_(OH)(_2−x_)xFx] and calcium fluoride (CaF_2_) both in the bulk phase and on HAP surfaces. Previous techniques calculate fluoride substitution as an average, rather than only of the newly precipitated material.

Previous work has been carried out using ^19^F MAS-NMR to follow FAP formation in fluoride-containing nano HAP toothpaste and to follow the conversion of fluoride-containing bioactive glasses used as toothpaste to FAP (25, 26) and as components of orthodontic adhesives (27), fluoride varnishes (28), air abrasives (29) and composites (27). In the case of fluoride-containing bioactive glasses, the fluorine present in the bioactive glass before undergoing a reaction can be readily distinguished from both fluorides in FAP and CaF_2_.

Thus, the primary aim of this study was to better understand the role of the interactions between fluoride and HAP minerals in established model systems for dental caries using ^19^F MAS-NMR and to utilise ^19^F MAS-NMR to further demonstrate the suitability of HAP as an enamel analogue in model enamel demineralisation studies by directly comparing the HAP data with the previously published enamel study by Mohammed et al. (15).

## 2. Materials and methods

### 2.1. Hydroxyapatite discs

Porous HAP discs with a nominal 20% porosity, measuring 12 mm ×  2 mm (D × H), were obtained from Plasma Biotal (Derbyshire, United Kingdom) and used as supplied.

### 2.2. Demineralisation of specimens

A series of fluoride-containing demineralising solutions were prepared from analytical grade reagents (Merck, Germany). A 10 L batch of demineralising solution of 0.1 M acetic acid buffered with potassium hydroxide to pH 4.0 was prepared with deionised water and subsequently divided into 0.5 L stock reservoirs. Sodium fluoride was added to the stock solutions to prepare concentrations containing 0, 1.0, 10, 25, 50, 100, 362.5, 750 and 1,450 ppm of [F^−^], selected to include concentrations of topically available fluoride. Calcium and phosphate were not used in the demineralisation solutions to keep the composition consistent with the previous study (15).

### 2.3. Mineral weight loss

Each HAP disc was placed in a container with 50 ml of demineralising solution (containing F^−^) at 37°C ± 1.0°C in a shaking incubator (KS 4000i control; IKA, United Kingdom) at 60 rpm for 96 h. Samples were dried and weighed using an analytical balance with an accuracy of ±0.1 mg (Mettler HK, Switzerland) before and after immersion to calculate the percentage mineral weight loss of each sample after 96 h.

### 2.4. ^19^F MAS-NMR

^19^F MAS-NMR spectra were obtained both before and after each HAP disc sample was immersed in 0.1 M acetic acid demineralising solutions containing the different [F^−^]. Each disc was dried after immersion and ground to a fine powder for a solid-state ^19^F MAS-NMR analysis. ^19^F MAS-NMR was carried out using a 600 MHz (14.1 T) spectrometer (Bruker, Germany) at a Larmor frequency of 564.5 MHz under spinning conditions of 15 kHz in a 2.5 mm rotor. The spectra were acquired using a low-fluorine background probe in a single-pulse experiment with a recycle duration of 30 s. The ^19^F chemical shift scale was referenced using the −120 ppm peak of 1 M sodium fluoride solution, with a secondary reference of trichlorofluoromethane. Typically, spectra were acquired for 1–17 h depending on the fluoride level and were an accumulation of 128–2,048 scans.

### 2.5. Determination of Ca, P and Na in the solution by ICP-OES

After the reaction with HAP discs, each fluoride-containing demineralising solution was analysed quantitatively for total calcium, phosphorus and sodium concentrations by ICP-OES (Varian Vista-PRO; Varian Ltd., United Kingdom). Each measurement was performed twice. Calcium, phosphorus and sodium standards of concentrations 0, 0.2, 0.5, 1, 2, 5, 10, 15, 20, 40, 60, 80 and 100 ppm were used. The instrumentation and experimental errors were determined by the difference between the two repeat measurements. The sodium values were matched to the [F^−^] up to 1,450 ppm.

### 2.6. Determination of [F^−^] before and after immersion and F uptake

The fluoride concentration before immersion and after immersion was determined using a fluoride ion-selective electrode (ISE; Nico2000 Ltd., United Kingdom). The difference in the values was then reported as the fluoride uptake by the discs from the solution.

## 3. Results

### 3.1. Solid-state ^19^F MAS-NMR

**Figure 1:**
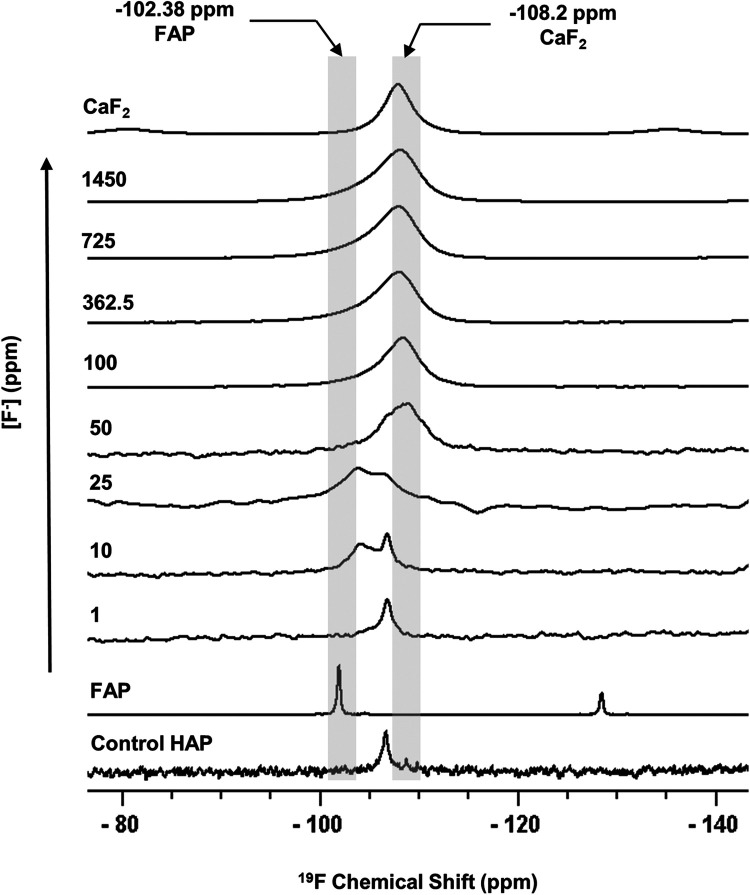
^19^F MAS-NMR spectra of HAP samples immersed in pH 4.0 demineralising fluoride solutions for 96 h. Control HAP spectra are of a disc that was not demineralised. Solutions of eight [F^−^] concentrations were used: 1, 10, 25, 50, 100, 327.5, 725, and 1,450. Two reference spectra are shown: FAP has a chemical shift between −102 and −103.8 ppm and CaF_2_ has a chemical shift between −107.5 and −108.5 ppm. Asterisks (*) mark spinning sidebands. MAS-NMR, magic angle spinning nuclear magnetic resonance; HAP, hydroxyapatite; FAP, fluorapatite.

Figure 1 shows the ^19^F MAS-NMR spectra of the HAP discs demineralised in the presence of a range of [F^−^]. The ^19^F MAS-NMR spectrum was also obtained for the HAP disc exposed to no F^−^, which was expected to exhibit a flat baseline with no detectable fluoride present; however, it exhibited a weak peak at −106.7 ppm. The FAP reference spectrum showed a characteristic peak in the range of −102.0 to −103.8 ppm corresponding to the F-Ca(3) sites in the FAP crystal structure, while the CaF_2_ reference spectrum showed a characteristic peak in the range of −107.5 to −108.5 ppm corresponding to the F-Ca(4) site. For the samples demineralised in the presence of concentrations of F^−^ below 25 ppm, the peak at −106.7 was always present.

### 3.2. Mineral weight loss

**Figure 2:**
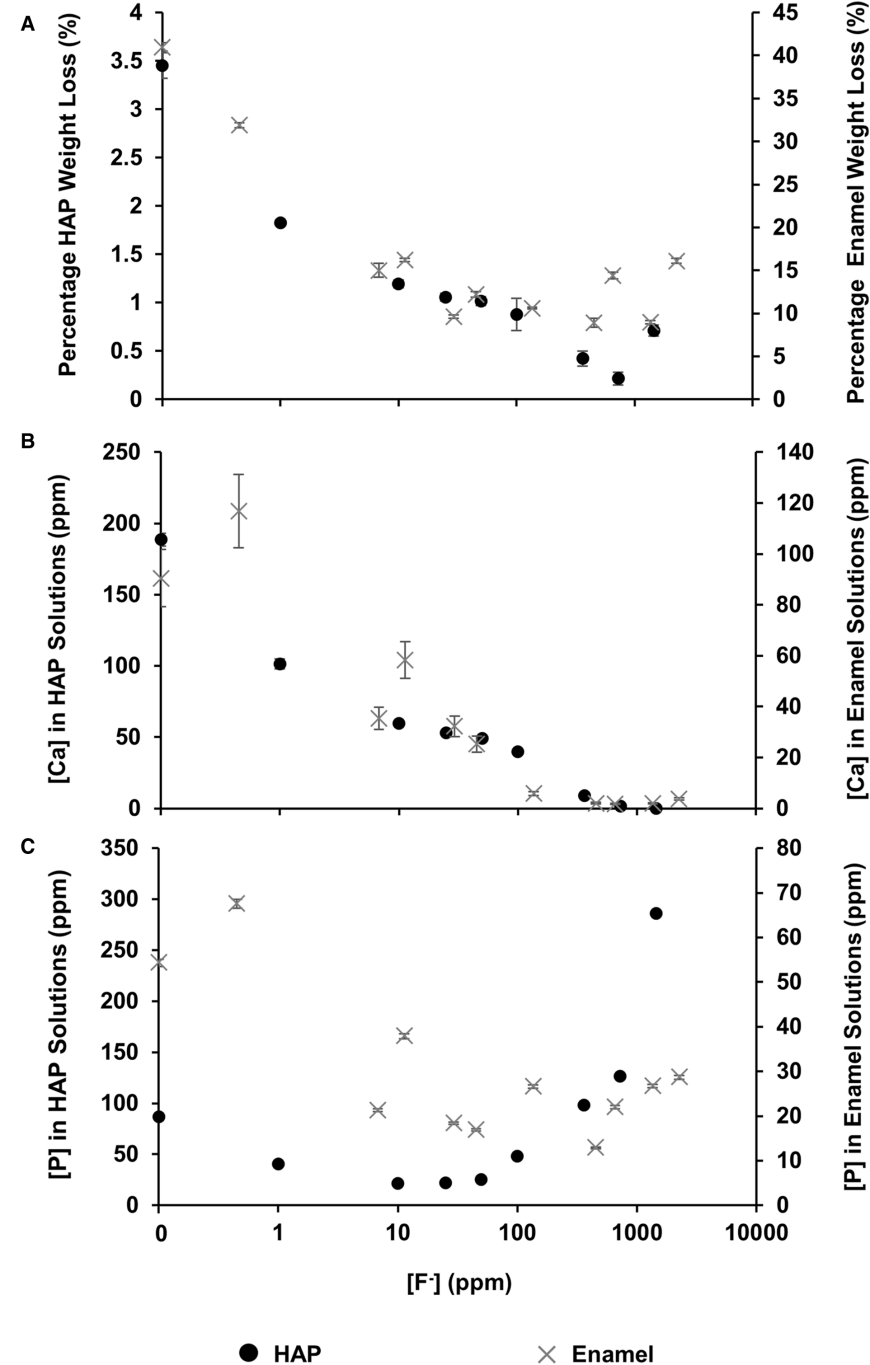
Data from this HAP study (left axis), as well as enamel data from the study by Mohammed et al. (15) (right axis), are presented. (**A**) The percentage weight loss is shown to reduce as fluoride concentration increases, (**B**) ICP-OES measurements for [Ca] and (**C**) ICP-OES measurements for [P] in solution, respectively, as a function of [F^–^] in the starting solution. ICP-OES, inductively coupled plasma optical emission spectroscopy.

The mineral loss was measured by comparing the percentage weight loss of the HAP disc samples before and after immersion in each of the demineralising solutions of different [F^−^]. Figure 2A shows that for the control HAP sample with no additional F^−^, there is a mean weight loss of 3.45% ± 0.13%. However, with increasing [F^−^] up to 725 ppm, the weight loss reduces to 0.22% ± 0.06%, whereas at 1,450 ppm, the weight loss increases to 0.71% ± 0.06%. Similar data for the previous enamel study are also shown for comparison.

### 3.3. ICP-OES

**Figure 3:**
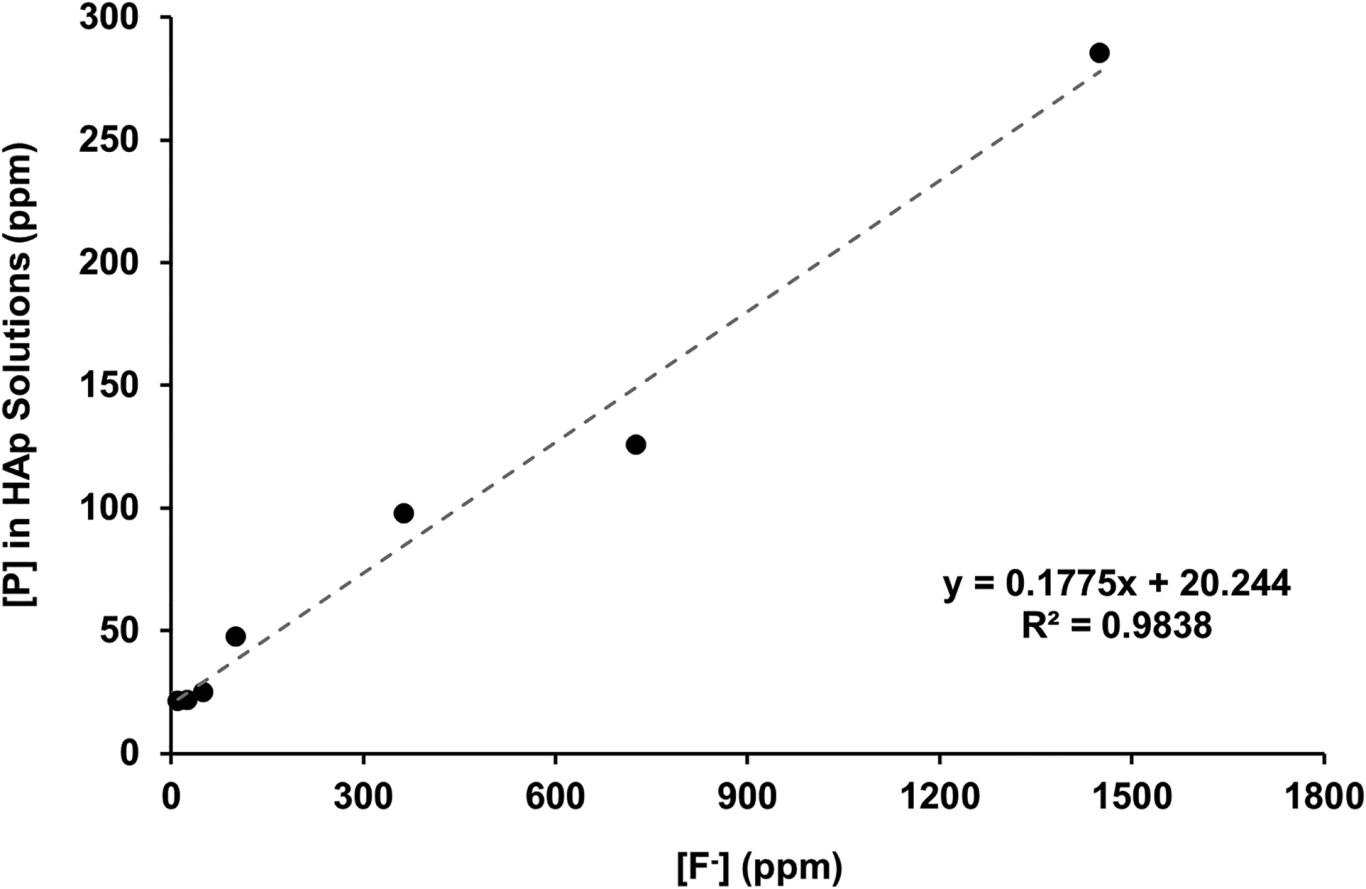
Linear correlation of [P] against [F^−^] in the starting solution above 10 ppm, measured with ICP-OES and fluoride ISEs, respectively. ICP-OES, inductively coupled plasma optical emission spectroscopy; ISE, ion-selective electrode.

Figure 2B shows the total calcium concentration and Figure 2C shows the total phosphorous concentration (and therefore phosphate ion) in the reaction solution following demineralisation of the HAP disc samples. For comparison, the data from the previous enamel study by Mohammed et al. (15) are also shown. For each substrate, both the calcium and the phosphate released into the demineralising solutions decreased markedly as the [F^−^] increased from 0 to 50 ppm. Further, the phosphate release was lower than the calcium release. Above 50 ppm [F^−^], the calcium concentration declined markedly, and the phosphate concentration increased significantly with increasing [F^−^], to values above the control at 0 ppm. Figure 3 shows the linear increase in phosphate concentration between 10 and 1,450 ppm [F^−^] treatment.

**Figure 4:**
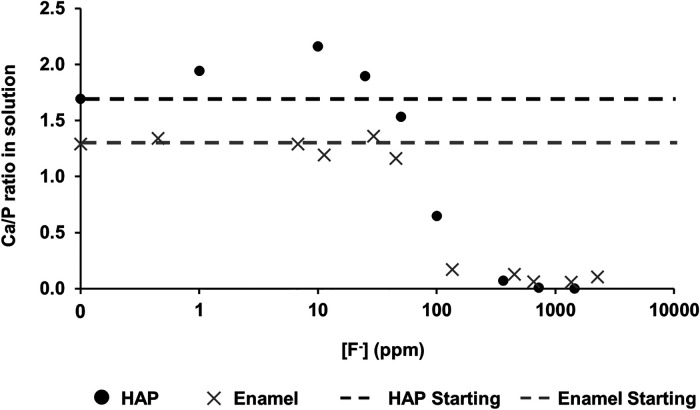
Ca/P ratio in the solution as a function of [F^–^] in the starting solution. Data from both this study (HAP) and that by Mohammed et al. (15) (enamel) are presented. HAP, hydroxyapatite.

Figure 4 shows the Ca:P ratio as a function of [F^−^] in the 0.1 M acetic acid solution for both HAP and enamel (previous study). For the control without fluoride, the Ca:P is 1.69 ± 0.20 close to the stoichiometry of HAP at 1.67. As the [F^−^] is increased to 100 ppm, the Ca:P ratio increases to 2.15 ± 0.01. Above this [F^−^], the Ca:P ratio declines markedly as the [F^−^] increases but declines only slightly above 362 ppm. The Ca:P ratio in the solution closely mirrors that of the previous enamel substrate study, except that the Ca:P ratio found for low [F^−^] <45 ppm is much lower for the enamel blocks, at about 1.2–1.4 compared with the stoichiometric ratio for HAP.

### 3.4. Fluoride concentrations before and after immersion

**Figure 5:**
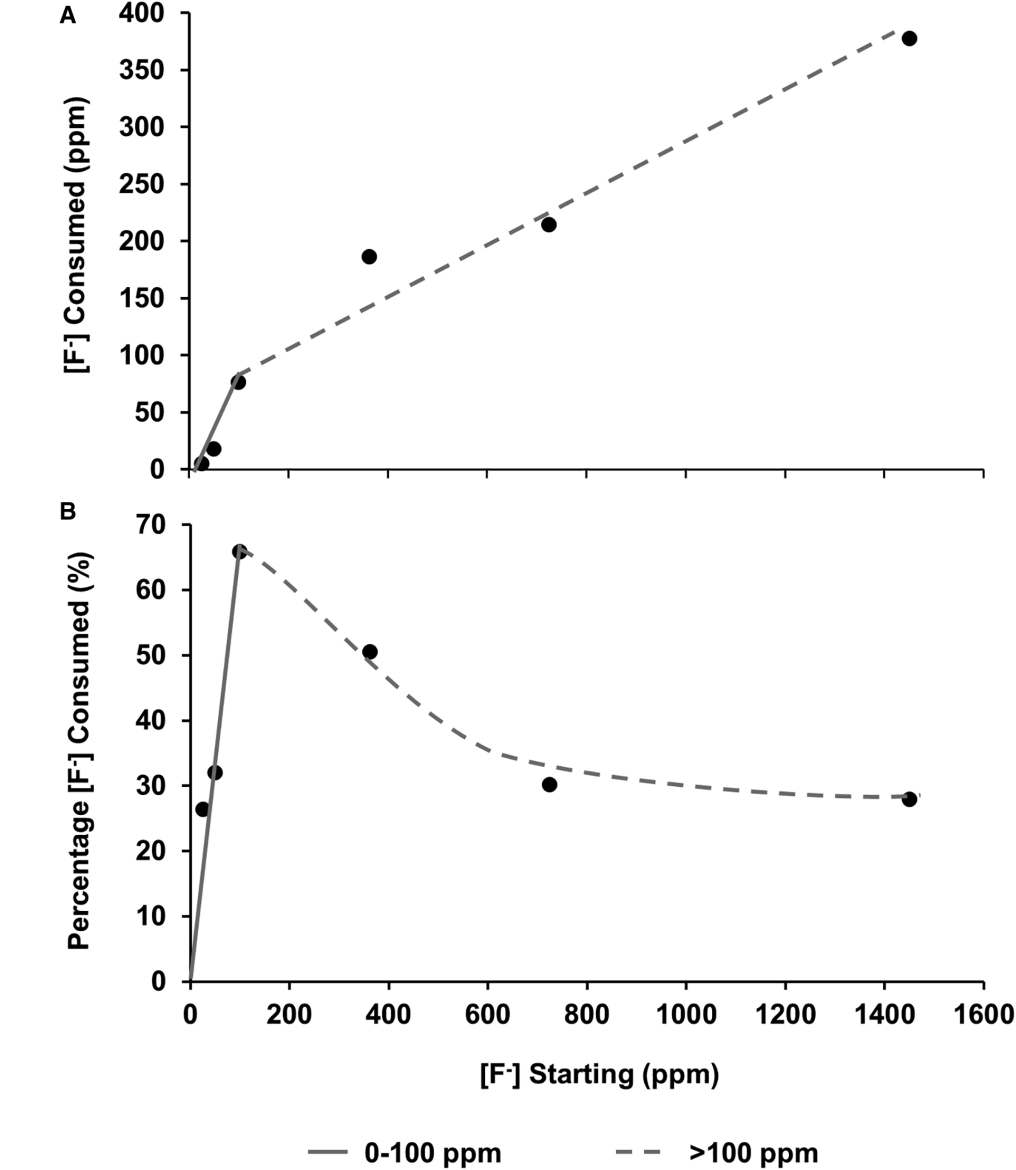
(**A**) fluoride consumed from the solution as a function of [F^–^] in the starting solution. (**B**) Percentage of [F^−^] consumed from solution by the HAP sample as a function of [F^–^] in the starting solution. From both data sets, it is hypothesised that there are two different behaviours at low (about 0-100 ppm) and high concentrations (above 100 ppm). HAP, hydroxyapatite.

The [F^−^] was also measured before and after immersion using a fluoride ion-selective electrode. After immersion with the HAP discs, the [F^−^] reduced. This is plotted as [F^−^] consumption (Figure 5A), as a percentage of fluoride available (Figure 5B), and as a function of the [F^−^] concentration in the demineralising solution and indicates the formation of fluoride-containing phases.

**Figure 6:**
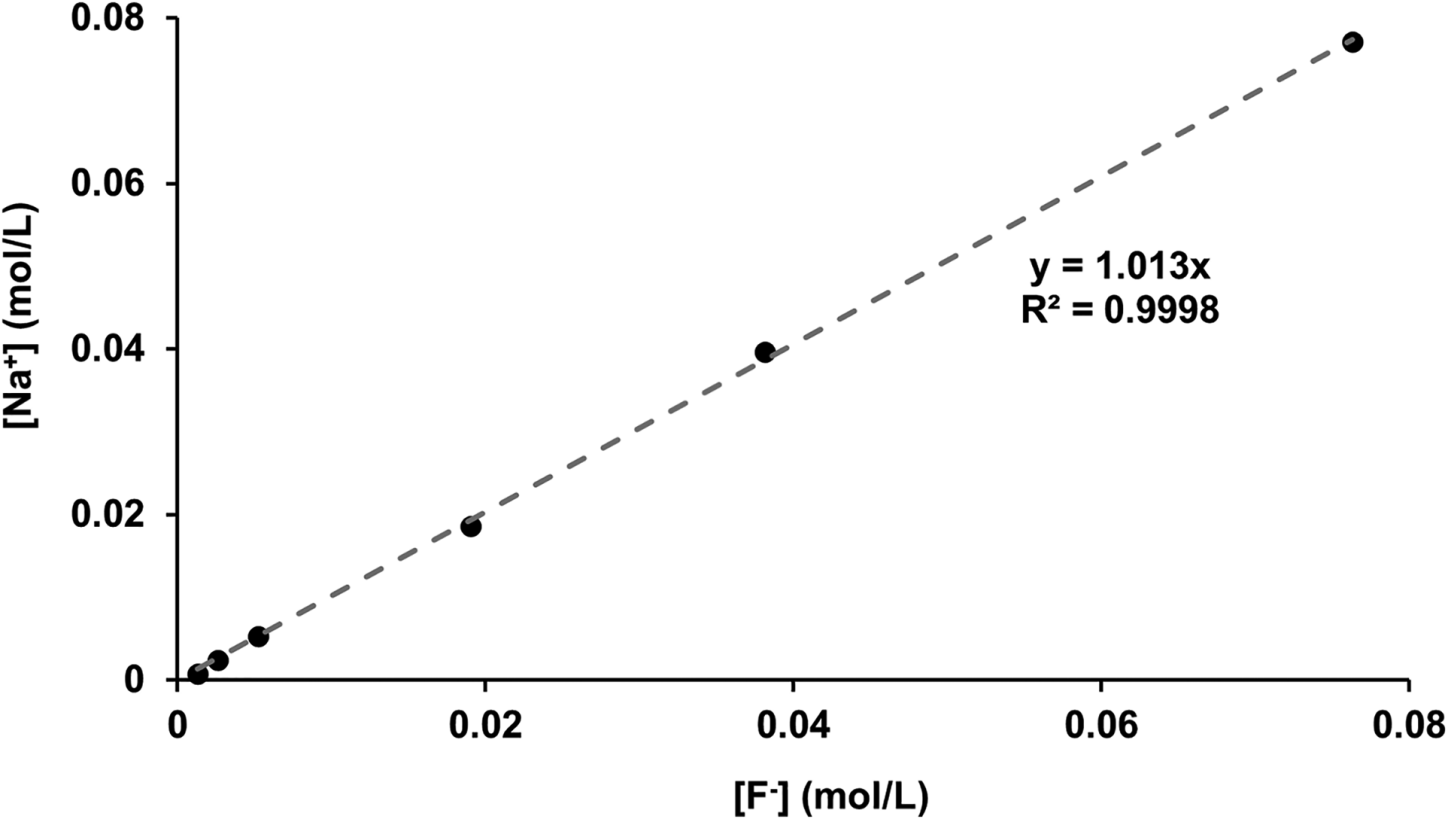
[Na^+^] measured with ICP-OES as a function of [F^−^] in the starting solution measured using fluoride ISEs. ICP-OES, inductively coupled plasma optical emission spectroscopy; ISE, ion-selective electrode.

For confirmation, the [F^−^] determined by ISE (before disc immersion) was plotted against the [Na] as determined by ICP-OES (shown in Figure 6). As expected, since the fluoride was added as NaF, there was a direct linear 1:1 correlation between [Na^+^] and [F^−^], corroborating the two measurement systems.

## 4. Discussion

The ^19^F peak at −106.7 ppm (Figure 1), plus the ISE detection of fluoride in the HAP disc on dissolution, indicates that the HAP disc does contain F^−^. However, the low concentration of F^−^ (0.145 ppm ± 0.065) released into the solution after demineralisation (HAP disc weight loss of 0.0232 g ± 0.0005) indicates a fluoride substitution for hydroxyl ions of between 1 and 3/100. The ^19^F chemical shift observed based on data by Gao et al. (18) indicates an F^−^ substitution of approximately 20%. Below this level of fluoride substitution, the ^19^F chemical shift no longer decreases with the decreasing F content of the HAP. This is probably because the observed F chemical shift is influenced by the first two adjacent OH groups in the c-axis channel of the crystal lattice but not by the third, and thus, the ^19^F chemical shift would not be expected to decrease any further after about one in five hydroxyl ions is substituted. That an ^19^F MAS-NMR signal can be detected for HAP containing such small amounts (0.03 wt. %) of fluoride demonstrates the exceptional sensitivity of the ^19^F MAS-NMR technique.

Based on the weight of HAP dissolved, the fluoride content of the hydroxyapatite was determined to be 0.84% ± 0.37% (this is the percentage of the OH groups substituted with F^−^). ^19^F chemical shifts were identified by comparison with the reference spectra for FAP, CaF_2_ and the plot of ^19^F chemical shift against F substitution in mixed FHAP from Gao et al. (18). The formation of heavily fluoride-substituted apatite [Ca_10_(PO_4_)_6_F(_2−x_) (OH)x where x≫0.1], i.e., close to FAP, was observed at an [F^−^] of 10 and 25 ppm. At an [F^−^] of 50 ppm, CaF_2_, in addition to FAP, was identified. Figure 2A shows that as the [F^−^] in the demineralising solution increased, the proportion of CaF_2_ increased and the proportion of FAP decreased. At 10 and 25 ppm [F^−^], there was evidence of a broad signal at about −105 ppm between that for FAP and the F in the original HAP, suggesting a FHAP solid solution. Upon immersion in acetic acid, the HAP discs demineralised, releasing calcium and phosphate into the solution. In the presence of F^−^, some of the calcium and phosphate reprecipitated as FAP, as evidenced by the peak at −103.7 ppm. In the case of the 10 ppm [F^−^], there is some evidence for a peak that is intermediate between −106.7 and −103 ppm that, based on Gao et al. (18), would correspond to a substitution of approximately 56% of F^−^ for OH^−^ in the HAP. It has been suggested that a mixed FHAP (Ca_10_(PO_4_)_6_F(2 − x) (OH)x, where x ≈ 1, demonstrates increased stability because of hydrogen bonding between the hydrogen of the hydroxyl groups and the fluoride ions in adjacent sites, as first reported in Moreno et al. (30) and later explained in Moreno et al. (31). The reprecipitation of apatite as FAP reduces both the weight loss and the concentration of Ca and P in the solution (Figure 2 shows the HAP data together with the enamel data from the previous study). As the F^−^ is smaller than the OH^−^, and is more electronegative, a reduction of the a-axis of the mixed FAP unit cell results (Aoba 1997). However, at an [F^−^] above 25 ppm, CaF_2_ forms in addition to FAP. The CaF_2_ formation consumes Ca^2+^ ions, which leaves excess PO_4_^3−^ ions in the solution above the apatite stoichiometry. Consequently, the P content in the solution increases and the Ca:P ratio decreases markedly. The formation of CaF_2_ above 45 ppm was also found by Mohammed et al. (15) in their studies on enamel, who also found P to increase in the solution and the Ca:P ratio to decline once CaF_2_ started to form.

Mohammed et al. (15) found that the Ca:P ratio in the solution was in the range of 1.2–1.4 (for studies on enamel), significantly lower than the ratio in stoichiometric hydroxyapatite of 1.67. However, natural enamel contains carbonate CO_3_^2−^, which is predominantly substituted by PO_4_^3−^ groups (B-type substitution) in the apatite lattice, resulting in a Ca-deficient apatite. This serves to reduce the Ca:P ratio in the solution. Carbonated HAP, as pointed out by Featherstone et al. (6), is more acid-soluble than HAP, and thus, the acid dissolution of hydroxy carbonate apatite results in carbonate elimination as well as the formation of FAP. The Ca:P ratio is also probably reduced in the solution because of the partial substitution of Ca^2+^ ions by other divalent ions, such as Mg^2+^ ions, in natural tooth enamel. There is an inverse relationship between carbonate and F content, via dissolution and reprecipitation of less highly carbonated apatite (32). In contrast, the Ca:P ratio found in the solution for the synthetic HAP is expectedly very close to the theoretical apatite stoichiometry.

The correlation between the measured [Na^+^] and the [F^−^] (Figure 6), plus the close matching of the values, confirms the validity of the ion-selective electrode measurements for fluoride as well as the accuracy of the ICP-OES sodium measurements (matching with the NaF added in grams). After immersion with the HAP discs, the [F^−^] decreased. The difference between the initial and the final [F^−^] represents the fluoride ions taken up to form both CaF_2_ FAP and fluoridated apatite.

Plotting the fluoride consumption as a percentage of the total added to the starting solution (Figure 5B) highlights the fact that the highest percentage consumption was at 100 ppm, which is also the point at which the concentration of the phosphate is higher than that of the calcium. A possible explanation may be that at the higher concentration of starting fluoride, the limiting factor is the demineralisation rate of the disc to release the calcium ions to take the fluoride out. This also helps explain the proposed reduction in the slope in Figures 5A, B once the [F^−^] is above 100 ppm, and the amount of CaF_2_ formation is dictating the percentage of fluoride consumed. On the other hand, at a lower [F^−^], it is the formation of FAP that is consuming the fluoride. There must be a transition range between FAP formation and CaF_2_ formation, which may be interesting to investigate in future work.

The concentration of phosphate released during demineralisation continued to increase between 10 and 1450 ppm of fluoride (Figure 3). This indicates that demineralisation is continuing and may even be driven by the Ca^2+^ ions removed from the solution as precipitated CaF_2_. Between the 362.5 and the 1450 ppm fluoride treatments, the phosphate concentration is higher (100–300 ppm) when compared with no fluoride treatment (<100 ppm), as shown in Figure 2C, demonstrating that high fluoride content in the solution can increase the amount of demineralisation.

Ren et al. (33) investigated the uptake of fluoride by HAP powder with a focus on removing F^−^ from contaminated water supplies as a function of [F^−^] at pH 7 and as a function of pH with 0.25 mM [F^−^]. They also used ^19^F MAS-NMR to study this process. F^−^ ions were taken up to form FAP at all pH values above pH 5, although it was thought to be an ion exchange process, rather than a dissolution and reprecipitation mechanism. At pH 7, they found CaF_2_ to form at an [F^−^] of 100 mM (1900ppm). At pH 4 with 0.25 mM [F^−^] (corresponding to 4.75 ppm), they also demonstrated the formation of fluorapatite. As the pH increased with immersion in 0.25 mM [F^−^], the full-width half maximum of the peak increased, indicating the possible formation of a mixed FHAP. The formation of FAP with 4.75 ppm [F^−^] is consistent with the results found in the present study.

The formation of CaF_2_ at an [F^−^] > 25 ppm was also found in a previous study (15) and supports the view of Featherstone et al. (6), Featherstone (34) and ten Cate (35) that it is low [F^−^] that is particularly beneficial in preventing demineralisation. It must be noted that in the studies reported here, CaF_2_ is forming under acidic conditions and CaF_2_ is exceedingly insoluble under acidic conditions (20, 21, 36) but soluble at pH > 9. Furthermore, there is no direct evidence for CaF_2_ formation at a low [F^−^] < 50 ppm (Figure 1). It is more likely that at these concentrations (<50 ppm), it is FAP, or even more likely, partially fluoridated apatite, that acts as a reservoir for fluoride, particularly if the crystallite size is small, which would favour their preferential dissolution compared with enamel apatite. However, Vogel et al. (37) reported no evidence for CaF_2_ formation *in vivo* following a rinse with NaF with fluoride concentrations at 228 ppm.

This suggests that the use of high fluoride prescription toothpaste is likely to result in CaF_2_ formation in the oral cavity. The benefit of such toothpastes is that it may maintain a higher [F^−^] for a longer period in the mouth in order to overcome the dilution effects that occur due to salivary flow *in vivo*. In both the present study and the previous study, there is no continuous model for salivary flow that results in fluoride clearance in the mouth (38–41) and, consequently, in the oral cavity, the [F^−^] drops quasi-exponentially with time, whereas the [F^−^] in the experimental model used in this study (ignoring the formation of fluoride-containing phases) is constant. Of course, the exact fluoride clearance process in the oral cavity will depend on salivary flow rates and salivary volumes, which will vary markedly from individual to individual. In addition, salivary flow decreases markedly at night. Furthermore, in the mouth, the pH would not remain close to pH 4.0 but would vary cyclically depending on the dietary habits of the individual. One of the major limitations of this study is that the experiments are performed statically in fixed immersion solutions, and *in vivo* there would be salivary flow and the pH would fluctuate rather than being constant. Further studies should include components to mimic salivary flow as well as a fluctuating pH.

## 5. Conclusion

This study has highlighted some key differences and similarities in the way that HAP discs and enamel respond to fluoride in acidic conditions. The HAP discs were seen to follow similar trends to those observed for the previous enamel data. However, the major advantage of using the standardised HAP discs was that there was less variability between specimens and, as such, trends were easier to distinguish. The HAP discs were found to contain fluoride as an impurity, and this is something that should be considered in future studies. The HAP discs were established as good enamel analogues for investigating the role of fluoride.

Further, this NMR study suggests that high levels of fluoride may accelerate demineralisation by taking calcium out of the solution in the form of precipitated calcium fluoride, thereby taking the system out of chemical equilibrium. Therefore, further work is required to determine the actual levels of fluoride required for therapeutic benefit, and in less undersaturated systems, and that also accounts for the effects of salivary flow rate, and fluctuating pH, using both HAP and enamel samples. In conclusion, under demineralising conditions, CaF_2_ formed on HAP at an [F^−^] of > 50 ppm, whereas fluoridated apatite formed below 50 ppm. The results were consistent with those obtained when an enamel substrate was used.

## Data Availability

The raw data supporting the conclusions of this article will be made available by the authors without undue reservation.
